# Comprehensive Characterization of Tumor Purity and Its Clinical Implications in Gastric Cancer

**DOI:** 10.3389/fcell.2021.782529

**Published:** 2022-01-10

**Authors:** Shenghan Lou, Jian Zhang, Xin Yin, Yao Zhang, Tianyi Fang, Yimin Wang, Yingwei Xue

**Affiliations:** ^1^ Department of Gastroenterological Surgery, Harbin Medical University Cancer Hospital, Harbin, China; ^2^ Department of Thoracic Surgery, Harbin Medical University Cancer Hospital, Harbin, China

**Keywords:** tumor purity, gastric cancer, prognosis, tumor microenvironment, chemotherapy resistance

## Abstract

Solid tumour tissues are composed of tumour and non-tumour cells, such as stromal cells and immune cells. These non-tumour cells constitute an essential part of the tumour microenvironment (TME), which decrease the tumour purity and play an important role in carcinogenesis, malignancy progression, treatment resistance and prognostic assessment. However, the implications of various purity levels in gastric cancer (GC) remain largely unknown. In the present study, we used an in-silico approach to infer the tumour purity of 2,259 GC samples obtained from our hospital and 12 public datasets based on the transcriptomic data. We systematically evaluated the association of tumour purity with clinical outcomes, biological features, TME characteristics and treatment response in GC. We found that tumour purity might be a patient-specific intrinsic characteristic of GC. Low tumour purity was independently correlated with shorter survival time and faster recurrence and significantly associated with mesenchymal, invasive and metastatic phenotypes. Integrating GC purity into a clinical prognostic nomogram significantly improved predictive validity and reliability. In addition, low tumour purity was strongly associated with immune and stromal cell functions. Fibroblasts, endothelial cells and monocytes were markedly enriched in low-purity tumours, serving as robust indicators of a poor prognosis. Moreover, patients with low GC purity may not benefit more from adjuvant chemotherapy. Our findings highlight that tumour purity confers important clinical, biological, microenvironmental and treatment implications for patients with GC. Therefore, a comprehensive evaluation of tumour purity in individual tumours can provide more insights into the molecular mechanisms of GC, facilitate precise classification and clinical prediction and help to develop more effective individualised treatment strategies.

## 1 Introduction

Gastric cancer (GC) is the fifth most prevalent cancer and the third most frequent cause of cancer-related deaths worldwide (Bray et al., 2018), with almost 1,000,000 new cases and 800,000 deaths each year (Torre et al., 2015; Bray et al., 2018). Owing to a lack of symptoms in the early stage, most patients with GC are usually diagnosed at an advanced stage (Dai et al., 2019). Treatment options for patients at an advanced stage are limited, resulting in a relatively low 5-year survival rate (<20%) (Katai et al., 2018). GC is a heterogeneous disease (Han et al., 2015), and different histopathological and molecular classification systems have been reported for its diagnosis (Serra et al., 2019). However, despite the widespread clinical use of histopathological classification (such as Lauren classification), the currently available histopathological systems remain insufficient to guide precise treatment for individual patients (Dicken et al., 2005). To date, the tumour–node–metastasis (TNM) staging system is considered the gold-standard method to predict prognosis and guide treatment decisions for patients with GC (Sasako et al., 2010). However, the high heterogeneity of GC leads to different outcomes among patients with the same TNM stage receiving the same treatments (Tang et al., 2020), suggesting that clinical prediction and treatment outcomes are unsatisfactory.

Solid tumour tissue comprises cellular components originating from various cancerous and noncancerous tissues, including immune, stromal, endothelial and epithelial cells (Joyce and Pollard, 2009). Such these noncancerous cells form an important part of the tumour microenvironment (TME) and interact with each other and tumour cells to sustain tumour growth and survival (Aran et al., 2015). GC tissues contain abundant GC-associated noncancerous cells within their microenvironments. With the increased understanding of the diversity and complexity of the TME of GC, these noncancerous cells, represented by stromal and immune cells, have been found to play an important role in carcinogenesis, malignancy progression and treatment resistance (Junttila and de Sauvage, 2013; Zeng et al., 2019; Zhang et al., 2020). However, the currently available classification systems merely consider the noncancerous factors present within GC tissues, and there is limited knowledge regarding the characteristics of GC cells under various purity levels.

Tumour purity, defined as the proportion of cancer cells in the tumour tissue, can be estimated by expert pathologists who review tumour sections or based on computational methods (Haider et al., 2020). However, pathological assessment may be inconsistent because of human error and bias (Smits et al., 2014). More importantly, tumour sections reviewed by pathologists may not always represent the tumour region that is subject to molecular profiling (Haider et al., 2020). However, estimation of tumour purity using alternative in-silico techniques can circumvent these problems. The continuously accumulating transcriptomic and genomic data provide an ideal resource for examining the multi-omic features underlying tumour purity in different cases. Integration of multiple independent studies is considered a better approach to enhancing the reliability of results, thus enabling us to identify the common core features of diseases (Ricketts et al., 2018).

Therefore, in the present study, we systematically evaluated the role of tumour purity in GC by integrating the clinical and multi-omic data of 2,259 GC samples obtained from our hospital, Gene Expression Omnibus (GEO) and The Cancer Genome Atlas (TCGA). We analysed the association of GC purity with clinical outcomes, functional characterisation and TME. In addition, we found that GC purity could predict the response of patients to chemotherapy. These findings provide novel insights into developing individualised treatment strategies for GC, which can help to improve prognostic risk stratification and facilitate treatment decision-making for patients with GC.

## 2 Materials and Methods

### 2.1 Gastric Cancer Dataset Source

Demographic information, clinical data and tissue samples were obtained from 214 patients with GC who had undergone gastrectomy as the primary treatment between 2016 and 2019 at the Harbin Medical University (HMU) Cancer Hospital. These data were used to construct the HMU-GC cohort. All samples were collected after written informed consent was obtained from the patients. This study was approved by the Institutional Review Board of the HMU Cancer Hospital. RNA isolation, library construction and mRNA sequencing were performed by Novogene (Beijing, China). The data were deposited in the GEO repository (GSE184336).

We systematically searched for publicly available GC gene expression datasets in GEO and TCGA databases. Datasets missing the follow-up data were excluded. In addition, to enhance the robustness of downstream analyses, samples with survival time less than 3 months were excluded (Joung and Merkow, 2021; Resio et al., 2021). A total of 12 public treatment-naive GC cohorts (GSE62254/ACRG, GSE15459, GSE57303, GSE34942, GSE38749, GSE15456, GSE84437, GSE26901, GSE26899, GSE13861, GSE26253 and TCGA-STAD) were selected for further analysis. In addition, four GC cell datasets, namely, Cancer Cell Line Encyclopedia (CCLE), GSE22183, GSE15455 and GSE146361, were included as references.

### 2.2 Data Preprocessing

To process microarray data, the raw CEL files obtained from Affymetrix were processed using the robust multichip average (RMA) algorithm for background correction and normalisation using the affy package (Irizarry et al., 2003; Gautier et al., 2004). The raw data from Illumina were processed using the limma package (Ritchie et al., 2015). For microarray datasets without raw data, the normalised matrix files were directly downloaded.

For high-throughput sequencing data obtained from the HMU-GC and TCGA-STAD datasets, raw read count values were converted to transcripts per kilobase million (TPM) values, which are more similar to those generated from microarrays and are comparable between samples (Wagner et al., 2012). Batch effects from non-biological technical biases among different datasets were corrected using the ComBat algorithm in the sva package (Leek et al., 2012).

The gene expression profile at the probe level (or Ensembl ID level) was converted to the official gene symbol level using the biomaRt package (Durinck et al., 2005). When multiple probes (or Ensembl IDs) were mapped to the same gene symbol, the probe (or Ensembl ID) with the largest mean expression values across samples was selected.

### 2.3 Identification of GC Purity and a Purity-Related Co-expression Network

Tumour purity was calculated using the ESTIMATE algorithm (Yoshihara et al., 2013). In addition, GC tissues in the TCGA-STAD cohort were reviewed to infer GC purity based on visual evaluation of the whole-slide images of haematoxylin and eosin (H&E) staining. Two pathologists independently confirmed the results of histological purity.

Weighted correlation network analysis (WGCNA) was performed using the WGCNA package to screen for purity-related gene modules (Langfelder and Horvath, 2008). A scale-free topology fitting index (R^2^) of 0.85 was set as the threshold to construct a signed weighted gene co-expression network. The minimum co-expression module size was set as 30, and the minimum cut height for module merging was set as 0.25. A biweight midcorrelation coefficient (R) > 0.4 and *p*-value < 0.05 were set as thresholds to screen for gene modules significantly associated with GC purity.

### 2.4 Association of GC Purity With Clinical, Molecular and Prognostic Features

Nearest template prediction (NTP) analysis was performed to classify samples based on the known clinical and molecular features (Hoshida, 2010). Univariate and multivariate Cox regression analyses were performed to calculate the hazard ratio (HR) and 95% confidence interval (CI). The Kaplan–Meier survival analysis with log-rank test was used to compare the survival rates of patients in different subgroups. Differences in prognosis between the high- and low-purity subgroups were compared using the restricted mean survival time (RMST) analysis (Kim et al., 2017). In addition, subgroup analyses were performed to examine the association between GC purity and other clinical characteristics.

Furthermore, GC purity was integrated with other independent prognostic factors to generate a composite prognostic nomogram for model visualisation and evaluation of clinical applications. The predictive value of the nomogram was compared with that of the TNM staging system in terms of concordance index (C-index). The performance of the composite model was evaluated based on calibration curves, time-dependent receiver operating characteristic (ROC) analysis, and decision curve analysis (DCA) (Vickers and Elkin, 2006).

### 2.5 Functional and Pathway Enrichment Analyses

Gene annotation enrichment analysis was performed using the clusterProfiler package (Yu et al., 2012). Gene set enrichment analysis (GSEA) was performed to screen for biological processes related to GC purity (Subramanian et al., 2005), and gene set variation analysis (GSVA) was performed to quantify the pathway enrichment scores using the GSVA package (Hänzelmann et al., 2013) and screen for significantly enriched pathways in each cluster. The well-defined ‘Hallmark gene sets’ were selected to quantify the pathway activity (Liberzon et al., 2015).

### 2.6 Estimation of Infiltrating Cells in the GC Microenvironment

To quantify the infiltration level of stromal and immune cells in each GC sample, we calculated the stromal and immune scores using the ESTIMATE algorithm (Yoshihara et al., 2013). Based on the guidelines of transcriptome-based cell-type quantification methods (Sturm et al., 2019), we used the MCPcounter and xCell algorithms to quantify specific immune and stromal cells in the GC samples (Becht et al., 2016; Aran et al., 2017). Based on the whole-slide images of H&E staining obtained from the TCGA-STAD cohort, we further used the convolutional neural network, a supervised deep-learning approach, to identify the proportion of tumour-infiltrating lymphocytes (TILs) in digitised H&E-stained tissue specimens (Saltz et al., 2018).

### 2.7 Estimation of the Potential Response to Chemotherapy

Seven common chemotherapeutic agents (5-fluorouracil, cisplatin, oxaliplatin, capecitabine, paclitaxel, docetaxel and irinotecan), which are approved for GC treatment, were selected to predict the chemotherapeutic response. Based on two public drug sensitivity databases, namely, CTRP (Basu et al., 2013) and PRISM (Corsello et al., 2020), the chemotherapeutic response was predicted via the pRRophetic package using ridge regression to estimate the area under the curve (AUC) value of each sample (Geeleher et al., 2014). Lower AUC values indicated increased sensitivity to treatment. The prediction accuracy was evaluated *via* 10-fold cross-validation based on each training set. Default values were selected for all parameters, including the combat algorithm for removing batch effects and the mean value for summarising duplicate gene expression.

### 2.8 Statistical Analyses

Differences between groups for continuous variables were evaluated using the Kolmogorov–Smirnov, Mann–Whitney or Kruskal–Wallis tests. The two-sided Pearson’s chi-squared or Fisher exact test was used to analyse the categorical data. The association between continuous variables was tested using Spearman correlation analysis. Restricted cubic splines were used to test potential non-linear associations. All statistical analyses were conducted using the R software, and *p*-values were two-sided. A *p*-value < 0.05 was considered statistically significant. The Benjamini–Hochberg method was used to control the false discovery rate (FDR) for multiple hypothesis testing.

## 3 Results

### 3.1 Tumour Purity Is an Intrinsic Property of GC

More than 40 GC cell lines involving 215 cell samples were analysed as references (Supplementary Table S1). A high degree of purity, i.e. median purity of 0.999 (inter-quartile range [IQR], 0.996–1), was observed in GC cell lines. Furthermore, no significant difference was found between the microarray and RNA-seq datasets (Kolmogorov–Smirnov test, *p* = 0.789), which verified the validity and robustness of the ESTIMATE algorithm.

**FIGURE 1:**
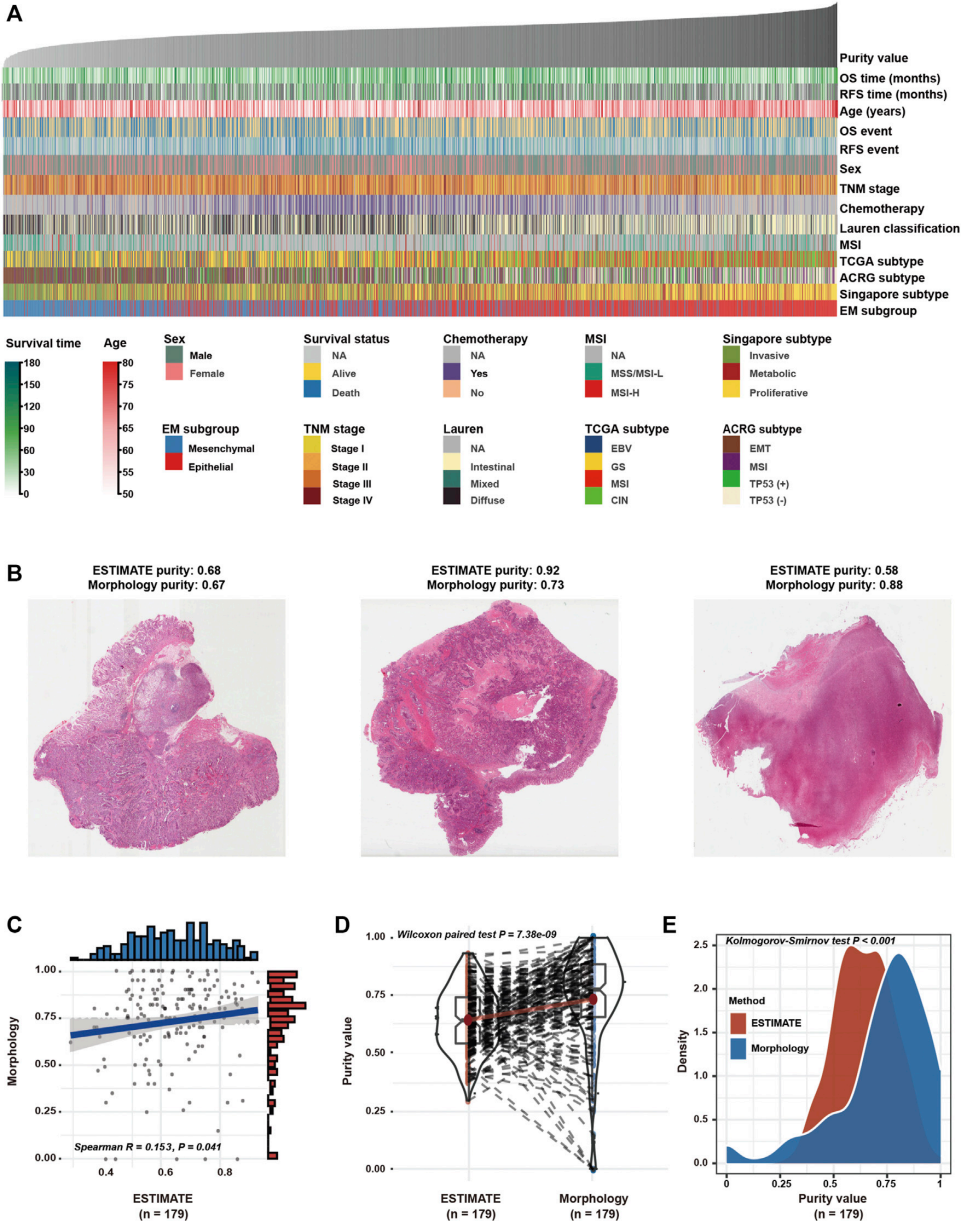
Tumour purity is an intrinsic property of gastric cancer (GC). **(A)** The landscape of clinicopathological and molecular characteristics associated with GC purity. **(B)** Representative slides of GC tissues. **(C)** Spearman correlation analysis of GC purity based on morphological assessment and the ESTIMATE method. **(D)** Distribution of GC purity evaluated based on morphological assessment and the ESTIMATE method. The upper and lower ends of the boxes represent the inter-quartile range of values. The lines in the boxes represent the median value. The whisker edges are the last data points within 1.5 of the inter-quartile range. The horizontal width of the violin represents the data density. **(E)** Density distribution of GC purity estimated based on morphological assessment and the ESTIMATE method.

In the GC meta-dataset (Supplementary Figure S1A), a total of 2,599 GC tissues from 13 cohorts were initially selected. After excluding samples with survival time less than 3 months (Joung and Merkow, 2021; Resio et al., 2021), 2,259 samples were retained for further analyses. As demonstrated in Figure 1A, GC samples were arranged in order of increasing purity, and a wide range (0.215–0.964) of purity was observed among the 2,259 samples, with a median purity of 0.681 (IQR, 0.608–0.759). In addition, the morphological features of GC cells were assessed in 179 patients in the TCGA cohort (Figure 1B; Supplementary Table S2). Consistent with the findings of the previous study (Aran et al., 2015), GC purity evaluated based on the ESTIMATE algorithm was significantly correlated with morphology (Figure 1C). However, significant differences were observed between the two evaluation methods (Figures 1D,E), which suggested that morphology evaluation can only provide a qualitative estimation of GC purity.

Furthermore, we investigated different samples from the same patient to determine their consistency. In the GSE14209 dataset, 22 patients were analysed twice (pre- and post-chemotherapy). We observed high concordance between the pre- and post-chemotherapy samples (Supplementary Figure S1B). The results suggest that tumour purity levels among patients with cancer are robust and consistent, implying that tumour purity may be an intrinsic property of GC.

### 3.2 GC Purity Is Characterised by Specific Clinicopathological and Molecular Features

**FIGURE 2:**
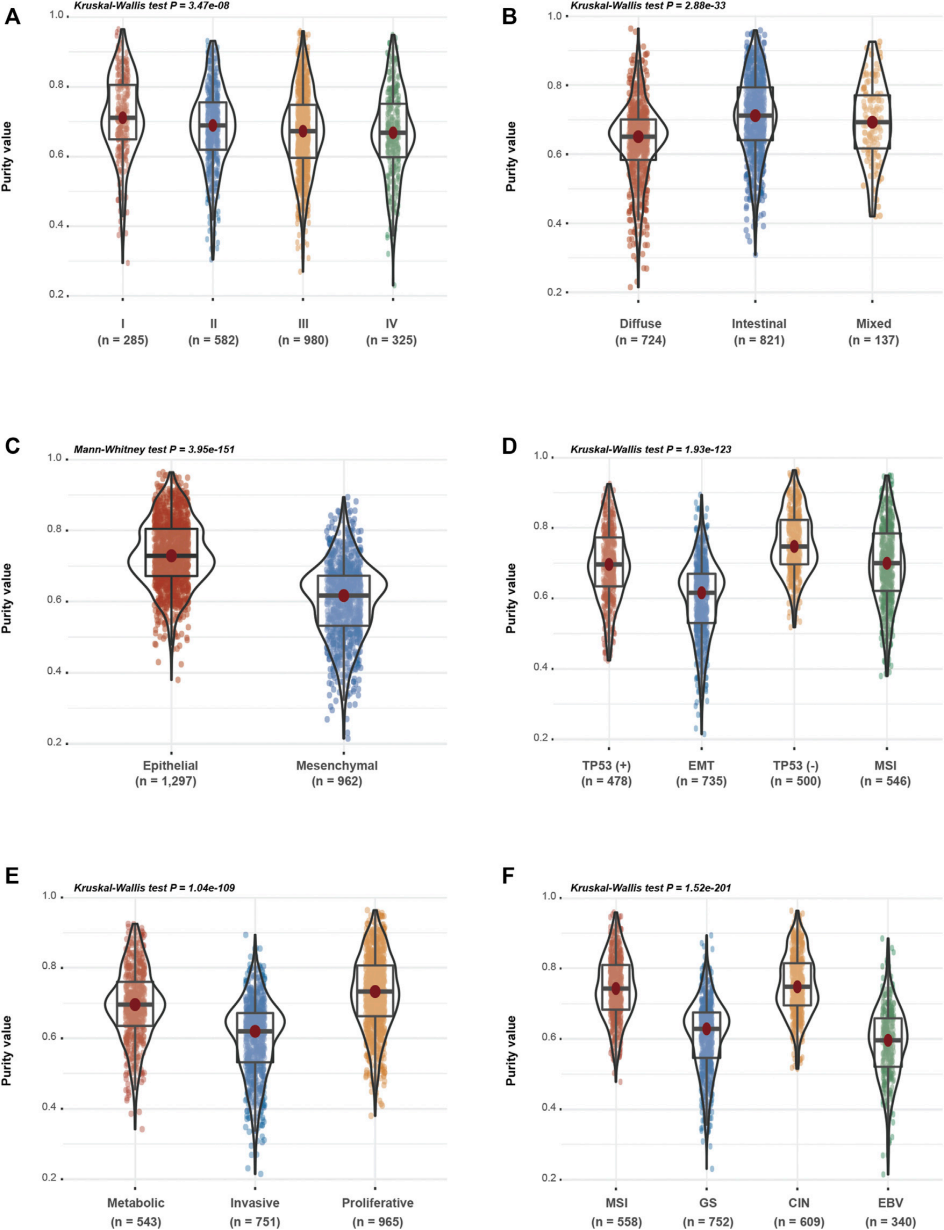
Gastric cancer (GC) purity is characterised by specific clinicopathological and molecular features. **(A–F)** Distribution of GC purity in terms of **(A)** TNM stage, **(B)** Lauren classification, **(C)** EM (epithelial and mesenchymal) subtype, **(D)** ACRG subtype, **(E)** Singapore subtype and **(F)** TCGA subtype. The upper and lower ends of the boxes represent the inter-quartile range of values. The lines in the boxes represent the median value. The whisker edges are the last data points within 1.5 of the inter-quartile range. The horizontal width of the violin represents the data density.

Based on the speculation that tumour purity is an intrinsic property of GC, we assessed the association between GC purity and clinical and molecular features. We found a negative correlation between GC purity and age at diagnosis (Spearman R = 0.122; *p* < 0.001; Supplementary Figure S1C). According to the TNM staging system and histopathological features, GC samples were classified into the corresponding clinicopathological and molecular features. A consistent decrease in GC purity was observed as the TNM stages advanced (Figure 2A). Based on the Lauren classification, intestinal-type GC had the highest purity level, whereas diffuse-type GC was accompanied had the lowest purity level (Figure 2B). These results suggest that low tumour purity is closely associated with the malignant progression of GC.

Based on clinical features, low-purity GC samples were more likely to belong to the mesenchymal subtype, whereas high-purity GC samples were enriched in the epithelial subtype (Figure 2C). Based on the other three molecular classifications, namely, the ACRG (Figure 2D), Singapore (Figure 2E) and TCGA (Figure 2F) classification systems, decreased tumour purity levels were associated with epithelial–mesenchymal transition (EMT) and invasive or genomically stable (GS) molecular subtypes, which were usually correlated with the invasion and metastasis of GC. These findings indicate that GC purity is closely related to specific clinicopathological and molecular features.

### 3.3 GC Purity Confers Different Survival and Recurrence Outcomes

We found that low purity was significantly related to the malignant phenotype. To further evaluate the prognostic value of GC purity in combination with other clinical variables, we performed univariate and multivariate Cox proportional hazard regression analyses based on combined clinicopathologic variables in the GC meta-dataset cohort. In addition to chemotherapy history and TNM stage, which is a well-known prognostic factor, GC purity was identified as a significant predictor of overall survival (OS) and recurrence-free survival (RFS) based on the univariate analysis (Table 1).

**TABLE 1:** Univariate and multivariate cox analyses for tumor purity in gastric cancer.

	Overall survival						Recurrence-free survival					
	Univariate			Multivariate			Univariate			Multivariate		
	Samples	HR (95%CI)	*p* value	Samples	HR (95%CI)	*p* value	Samples	HR (95%CI)	*p* value	Samples	HR (95%CI)	*p* value
Age												
Increasing years	2254	1.014 (1.009, 1.019)	**<0.001**	772	1.002 (0.991, 1.012)	0.765	1155	1.003 (0.995, 1.011)	0.499			
Gender												
Female	764	Reference					400	Reference				
Male	1495	1.052 (0.922, 1.200)	0.449				757	1.045 (0.858, 1.273)	0.66			
Chemotherapy												
No	131	Reference		112	Reference		104	Reference		103	Reference	
Yes	732	0.497 (0.364, 0.678)	**<0.001**	660	0.337 (0.239, 0.475)	**<0.001**	556	0.290 (0.209, 0.403)	**<0.001**	556	0.243 (0.174, 0.338)	**<0.001**
Lauren classification												
Intestinal	821	Reference		328	Reference		541	Reference				
Mixed	137	1.521 (1.174, 1.970)	**0.001**	60	1.218 (0.780, 1.903)	0.386	60	1.505 (0.984, 2.303)	0.06			
Diffuse	724	1.152 (0.991, 1.340)	0.065	384	1.007 (0.773, 1.311)	0.962	505	1.128 (0.928, 1.370)	0.227			
TNM stage												
Stage I	285	Reference		129	Reference		193	Reference		125	Reference	
Stage II	582	1.579 (1.142, 2.182)	**0.006**	222	1.328 (0.806, 2.188)	0.266	375	1.303 (0.882, 1.924)	0.183	202	1.411 (0.886, 2.247)	0.147
Stage III	980	3.407 (2.527, 4.593)	**<0.001**	289	3.209 (2.035, 5.061)	**<0.001**	382	2.630 (1.819, 3.802)	**<0.001**	206	3.273 (2.120, 5.051)	**<0.001**
Stage IV	325	7.363 (5.378, 10.079)	**<0.001**	132	5.768 (3.599, 9.244)	**<0.001**	201	5.464 (3.752, 7.956)	**<0.001**	125	4.454 (2.862, 6.932)	**<0.001**
Tumor purity												
Increasing values	2259	0.455 (0.269, 0.771)	**0.003**	772	0.120 (0.025, 0.570)	**0.007**	1157	0.372 (0.158, 0.874)	**0.023**	659	0.129 (0.025, 0.663)	**0.014**

CI, confidence interval; HR, hazard ratio. The bold values mean that the results were statistical significance.

**FIGURE 3:**
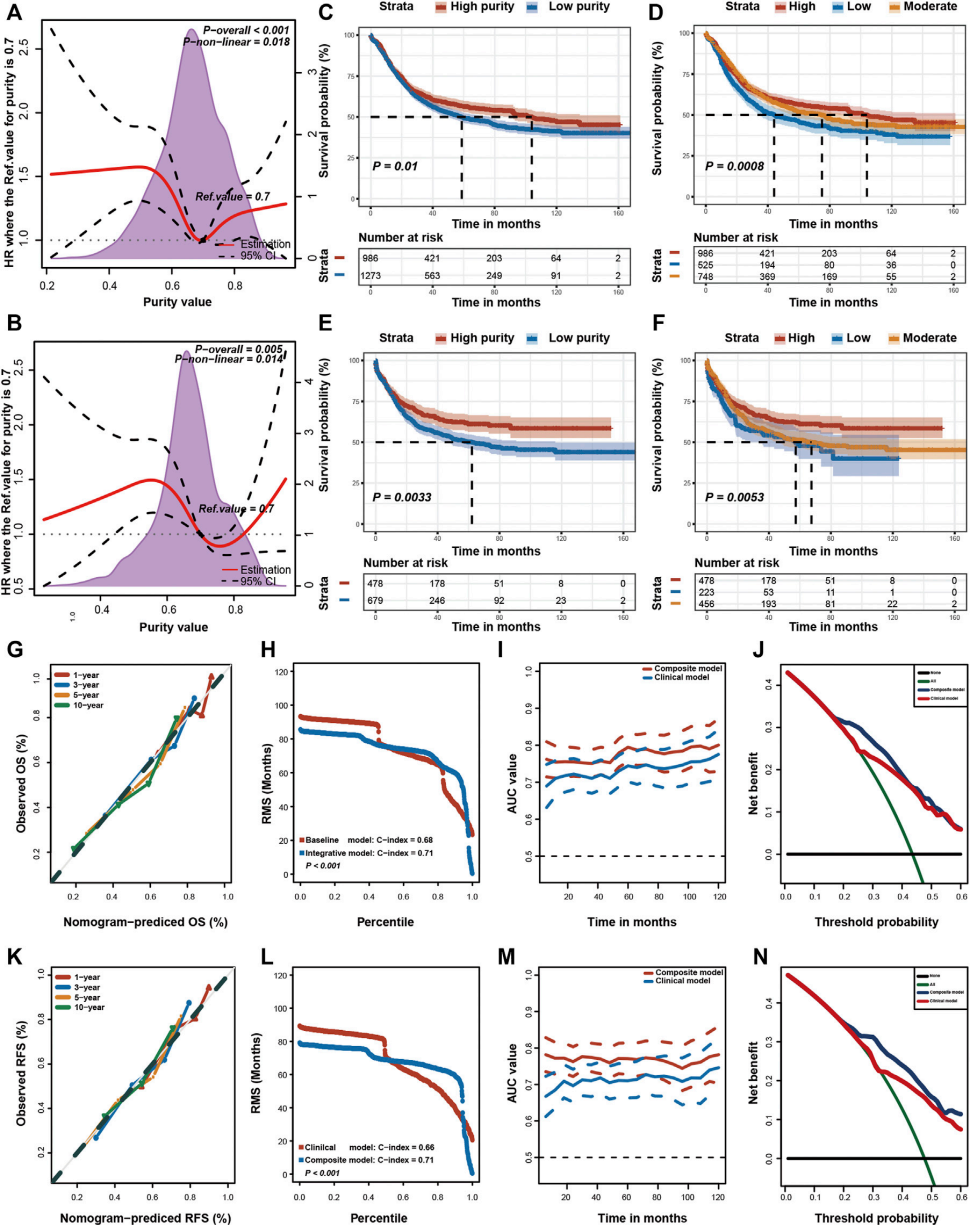
Prognostic value of tumour purity in gastric cancer. **(A,B)** Association of tumour purity and **(A)** overall survival (OS) and **(B)** recurrence-free survival (RFS) estimated using restricted cubic splines. **(C–F)** Kaplan–Meier curves for **(C,D)** OS and **(E,F)** RFS among different subgroups. *p*-values were obtained using the log-rank test. The + symbols in the panels indicate censored data. **(G,K)** Calibration plots of the nomogram for the predicted **(G)** OS and **(K)** RFS at 1, 3, 5, and 10 years. **(H,L)** Restricted mean survival (RMS) time curves for **(H)** OS and **(L)** RFS. **(I,M)** Time-dependent ROC curves for **(I)** OS and **(M)** RFS. **(J,N)** Decision curve analysis for the prediction of **(J)** OS and **(N)** RFS using the nomogram. HR, hazard ratio.

Although the Epstein–Barr virus (EBV) subtype is characterised by lower purity scores (Figure 2F), it is associated with a good prognosis (Sohn et al., 2017). Therefore, the relationship between GC purity and prognosis may not necessarily be linear. We used restricted cubic splines to assess the possible non-linear association between GC purity and prognosis. However, a non-linear relationship (a U-shaped curve) was observed between GC purity and OS (Figure 3A) and RFS (Figure 3B).

Furthermore, patients with GC were stratified into two (high or low purity) and three (high, moderate or low purity) subgroups using the optimal cut-off values determined using the X-tile software (Camp et al., 2004). In both subgroup analyses, the OS (Figures 3C,D) and RFS (Figures 3E,F) rates were lower in patients with low purity scores than in patients with high purity scores. The RMST difference also determined a benefit of high purity, and the benefit seemed to increase over time (Table 2). For example, the RMST differences between the two groups were 2 months for OS and 4 months for RFS after 5 years of follow-up, which increased to 7 months for OS and 12 months for RFS after 10 years. These results suggested that low GC purity was associated with a shorter survival time and faster recurrence.

**TABLE 2:** Restricted mean survival time (RMST) differences in different time points.

Overall survival										
Time point	High purity (*n* = 986)			Low purity (*n* = 1273)			RMST difference^a^			
	RMST	95% CI		RMST	95% CI		Effect size	95% CI		*p* value
12 months	11.096	10.938	11.253	11.009	10.862	11.156	0.087	−0.128	0.303	0.428
36 months	28.054	27.294	28.813	27.509	26.841	28.176	0.545	−0.466	1.557	0.291
60 months	42.216	40.742	43.689	40.361	39.091	41.632	1.855	−0.091	3.8	0.062
84 months	55.412	53.179	57.644	51.701	49.797	53.606	3.71	0.776	6.645	**0.013**
120 months	73.604	70.179	77.03	67.03	64.118	69.942	6.574	2.078	11.071	**0.004**
160 months^b^	91.961	86.98	96.942	83.132	78.979	87.285	8.829	2.344	15.314	**0.008**

Recurrence free survival										
Time point	High purity (*n* = 478)			Low purity (*n* = 679)			RMST difference^a^			
	RMST	95% CI		RMST	95% CI		Effect size	95% CI		*p* value
12 months	10.408	10.088	10.728	10.355	10.084	10.625	0.054	−0.365	0.472	0.802
36 months	27.384	26.131	28.637	25.688	24.633	26.744	1.695	0.057	3.333	**0.043**
60 months	42.541	40.22	44.862	38.472	36.529	40.416	4.069	1.042	7.096	**0.008**
84 months	57.137	53.67	60.604	50.045	47.174	52.916	7.092	2.591	11.594	**0.002**
120 months	78.252	72.882	83.621	66.409	62.032	70.787	11.842	4.914	18.77	**0.001**
150 months^b^	95.8	88.686	102.915	79.602	73.837	85.367	16.198	7.041	25.355	**0.001**

RMST, restricted mean survival time. The bold values mean that the results were statistical significance.

a RMST difference = RMST_high purity_−RMST_low, purity_.

b Final follow-up time point.

When all relevant clinical variables were included in the multivariate Cox regression analysis, GC purity was identified as a significant prognostic factor (Table 1). Subgroup analyses were further performed to assess the association between GC purity and other prognostic factors. No significant association was observed between GC purity and both OS and RFS (Table 3), indicating that GC purity retained its prognostic relevance even after classic clinicopathological prognostic features were considered.

**TABLE 3:** Subgroup analysis for tumor purity in gastric cancer.

	Overall survival			Recurrence free survival		
	Samples	HR (95%CI)	*p* value for interaction	Samples	HR (95%CI)	*p* value for interaction
Gender						
Female	764	**0.013 (0.001, 0.286)**	0.2	400	**0.001 (0.0001, 0.024)**	0.003
Male	1495	0.214 (0.035, 1.300)		757	0.590 (0.081, 4.288)	
**Age**						
<65	1461	**0.063 (0.009, 0.447)**	0.589	797	0.065 (0.008, 0.506)	0.4716
>65	793	0.274 (0.021, 3.627)		358	0.147 (0.009, 2.353)	
Chemotherapy						
No	131	0.201 (0.018, 2.301)	0.681	104	0.074 (0.006, 0.970)	0.7392
Yes	732	**0.100 (0.014, 0.706)**		556	0.148 (0.017, 1.320)	
Lauren type						
Intestinal	821	0.135 (0.018, 1.040)	0.849	541	0.062 (0.007, 0.522)	0.1588
Mixed	137	1.238 (0.012, 130.179)		60	42.089 (0.045, 39,734.047)	
Diffuse	724	0.140 (0.009, 2.252)		505	0.302 (0.014, 6.298)	
TNM stage						
Stage I	285	0.355 (0.009, 13.991)	0.076	193	0.017 (0.0004, 0.800)	0.3875
Stage II	582	0.002 (0.00003, 0.072)		375	0.004 (0.0001, 0.186)	
Stage III	980	0.572 (0.057, 5.730)		382	0.245 (0.013, 4.500)	
Stage IV	325	0.115 (0.005, 2.812)		201	0.291 (0.015, 5.804)	

HR, hazard ratio; CI, confidence interval. The bold values mean that the results were statistical significance.

Furthermore, we integrated these independent factors (tumour purity, TNM stage and chemotherapy history) to establish a prognostic nomogram for predicting the OS of patients with GC (Supplemenatry Figure S2A). The calibration plot revealed an optimal agreement between nomogram prediction and actual observation at different time points (Figure 3G). Moreover, we compared the OS prediction power between the nomogram and clinical model. The C-index of the nomogram was significantly higher than that of the clinical model for predicting OS (Figure 3H). In addition, the time-dependent ROC curve confirmed that the nomogram exhibited better performance in predicting the prognosis of GC (Figure 3I).

Lastly, DCA curves were used to assess the clinical usefulness and net benefit of these two models. The composite nomogram demonstrated a larger net benefit than that exhibited by the clinical model within most of the threshold probabilities (Figure 3J). Similar results were also found for RFS (Supplemenatry Figure S2B; Figures 3K–N). These results indicated that integrating GC purity into a prognostic model can significantly improve its predictive power. In addition, the nomogram exhibited better clinical utility for predicting prognosis, emphasising the requirement of considering GC purity in clinical practice.

### 3.4 Biological Insights Into GC Purity

**FIGURE 4:**
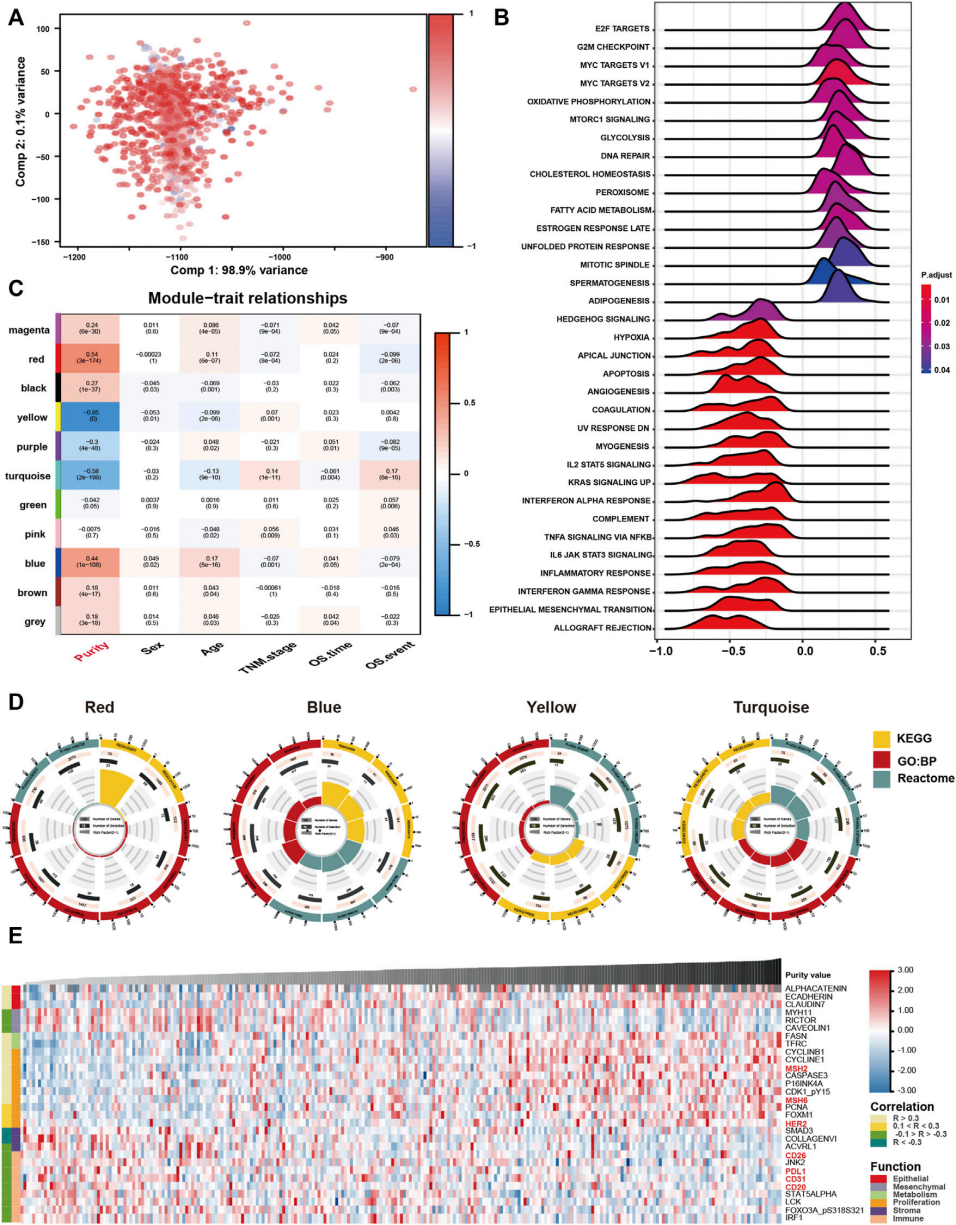
Biological features of tumour purity in gastric cancer. **(A)** Principal component analysis score plot for the gene expression profile underlying different purity levels. **(B)** Gene set enrichment analysis associated with purity levels. **(C)** Trait and module relationship analysis. Each row corresponds to a module eigengene and each column to a trait. The top number represents the biweight midcorrelation coefficient of each cell, and the corresponding *p*-values are mentioned in brackets. **(D)** Representative results of functional enrichment analysis for the yellow, blue, red and turquoise modules. **(E)** Heatmap of the reverse-phase protein arrays demonstrating purity-associated protein production. The coefficient was evaluated via Spearman correlation analysis.

Given that tumour purity is closely related to the clinicopathological, molecular and prognostic features, we used RNA expression profiles to examine the underlying biological processes associated with GC purity. We performed principal components analysis (PCA) to identify the transcriptomic features related to GC purity and found a strong association between mRNA expression profiles and GC purity (Figure 4A), implying that distinct biological phenotypes are attributed to varied GC cell percentages.

Furthermore, we performed GSEA to assess the biological features associated with GC purity. The results suggested that samples with different GC purity exhibited distinct biological processes (Figure 4B). Consistent with the clinical and molecular features, low tumour purity was significantly related to pathways associated with invasion and metastasis and multiple immune-related pathways. However, high purity was considerably associated with metabolism and proliferation-related pathways.

Subsequently, we used WGCNA to obtain purity-related modules. We constructed a cluster dendrogram according to the soft threshold power (Supplemenatry Figure S3A) and identified 11 colour modules (Supplemenatry Figure S3B). Among the identified modules, four were highly associated (|R| > 0.4) with tumour purity (Figure 4C; Supplemenatry Table S3). In addition, gene significance significantly correlated with module membership in each module (Supplemenatry Figure S3C), suggesting that genes in these modules might play an essential role associated with GC purity.

Furthermore, module enrichment analyses were performed to explore the biological features of purity-related modules (Figure 4D). Consistent with the results of GSEA, genes in the red module were enriched in pathways associated with metabolic activation (Supplemenatry Table S4). Genes in the blue module were significantly enriched in proliferation-specific pathways (Supplemenatry Table S5). In addition, genes in the turquoise (Supplemenatry Table S6) and yellow (Supplemenatry Table S7) modules were prominently related to stromal and immune activation pathways, respectively.

Eventually, we analysed proteomic data generated using reverse-phase protein arrays in the TCGA cohort (Figure 4E). Consistent with the clinical and gene expression data, *α*-catenin, E-cadherin and other crucial epithelial adhesion proteins in epithelial cells were positively correlated with tumour purity. Low tumour purity was associated with high expression of MYH11, RICTOR and CAV1, which are markers for mesenchymal lineage or EMT. In addition, low GC purity was associated with increased expression of numerous stromal- and immune-associated proteins. High GC purity was, however, associated with increased expression of multiple proliferation- and metabolism-associated proteins.

### 3.5 GC Purity is Associated With Infiltration of Distinct Stromal and Immune Cells

**FIGURE 5:**
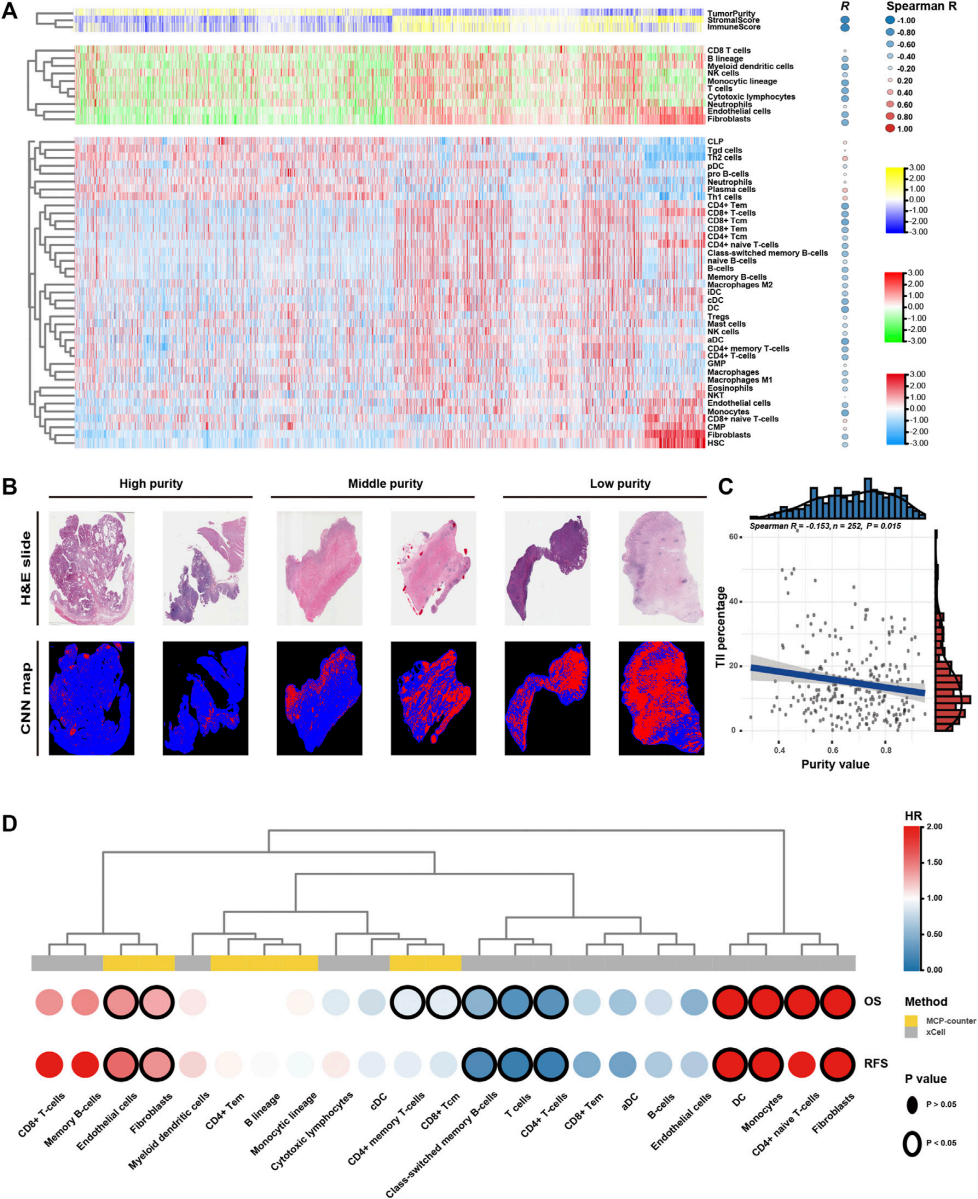
Tumour microenvironment features of tumour purity in gastric cancer (GC). **(A)** Heatmap of the infiltration level of stromal and immune cells among the purity-based subtypes. **(B)** Representative slides (top) and tumour-infiltrating lymphocyte (TIL) maps (bottom) of GC tissues with different purity values. The red colour represents a positive TIL patch, the blue colour represents a tissue region with no TIL patch and the black colour represents no tissue. **(C)** The Spearman correlation between TILs and purity value. **(D)** Heatmap of survival analysis of purity-related stromal and immune cells for overall survival (OS) and recurrence-free survival (RFS). CNN, convolutional neural network; H&E, haematoxylin and eosin; OS, overall survival; RFS, recurrence-free survival; HR, hazard ratio.

Low GC purity was markedly associated with stromal and immune activation pathways (Figure 4). Therefore, we further examined the relationship between TME features and GC purity to characterise TME heterogeneity. Both stromal and immune scores, representing the overall infiltration of stromal and immune cells, respectively, in tumour tissues, were inversely correlated with GC purity (Figure 5A), suggesting that TME with decreased tumour purity had a significantly increased infiltration of stromal and immune cells. In addition, TILs were confirmed to be negatively correlated with GC purity (Figures 5B,C) (Saltz et al., 2018).

Furthermore, we used the MCPcounter algorithm to evaluate the relative abundance of infiltrating stromal and immune cell subpopulations against GC purity (Figure 5A). T cells, cytotoxic lymphocytes, B lineage, myeloid dendritic cells, monocytic lineage, fibroblasts and endothelial cells exhibited a consistently negative correlation (R < −0.4) with GC purity. We further characterised TME heterogeneity using the xCell algorithm, which can evaluate as many as 39 different immuno-oncology cell types (Figure 5A). Similar to the previous result, fibroblasts, endothelial cells, myeloid dendritic cells and several T and B cell types were found to be negatively correlated with GC purity (R < −0.4). Although numerous immune cell types (CD8^+^ naive T cells, Th_1_ cells and Th_2_ cells) were positively associated with GC purity, the correlation was relatively weak.

Lastly, we attempted to assess the prognostic implications of these purity-related stromal and immune cells in GC (Figure 5D). Survival analyses revealed that fibroblasts and endothelial cells were consistently associated with worse OS and RFS. However, T cells, particularly memory T cells, were consistently associated with better OS and RFS. The results of the xCell algorithm revealed that monocytes might correlate with worse OS and RFS. Therefore, T cells, fibroblasts, endothelial cells and monocytes can be a cluster of nontumour cells contributing to TME and specific clinical outcomes for patients with GC with varying purity levels.

### 3.6 GC Purity is Strongly Correlated With Chemotherapy Response

Chemotherapy is the mainstay of treatment for patients with advanced GC. However, chemotherapy resistance is the primary cause of treatment failure. Given that distinct clinical, biological and microenvironmental features are associated with GC purity, we further investigated the relationship between GC purity and chemotherapy response to promote individualised treatment decisions. The analysis was performed in a subset of patients in the meta-data cohort with data on chemotherapy (*n* = 863). Of the 863 patients, 732 received adjuvant chemotherapy. We found that GC purity could serve as an independent predictor for adjuvant chemotherapy benefit (Table 4). Patients with GC with a high purity level who received adjuvant chemotherapy had a significant OS benefit (HR, 0.101; 95% CI, 0.014–0.719; *p* = 0.022).

**TABLE 4:** Univariate and multivariate cox analyses for tumor purity in patients who received adjuvant chemotherapy.

	Univariate			Multivariate		
	Samples	HR (95%CI)	*p* value	Samples	HR (95%CI)	*p* value
Age						
Increasing years	732	1.006 (0.996, 1.017)	0.22			
Gender						
Female	250	Reference				
Male	482	0.929 (0.731, 1.180)	0.545			
Lauren classification						
Intestinal	248	Reference	Reference	248	Reference	Reference
Mixed	46	1.783 (1.092, 2.911)	**0.021**	46	1.662 (1.013, 2.728)	**0.044**
Diffuse	366	1.253 (0.957, 1.640)	0.101	366	1.088 (0.827, 1.432)	0.548
TNM stage						
Stage I	84	Reference	Reference	79	Reference	Reference
Stage II	220	3.027 (1.443, 6.351)	**0.003**	204	2.681 (1.272, 5.653)	**0.01**
Stage III	294	7.258 (3.555, 14.819)	**<0.001**	263	6.581 (3.213, 13.480)	**<0.001**
Stage IV	134	14.248 (6.913, 29.369)	**<0.001**	114	11.932 (5.747, 24.777)	**<0.001**
Tumor purity						
Increasing values	732	0.066 (0.013, 0.343)	**0.001**	660	0.101 (0.014, 0.719)	**0.022**

HR, hazard ratio; CI, confidence interval. The bold values mean that the results were statistical significance.

**FIGURE 6:**
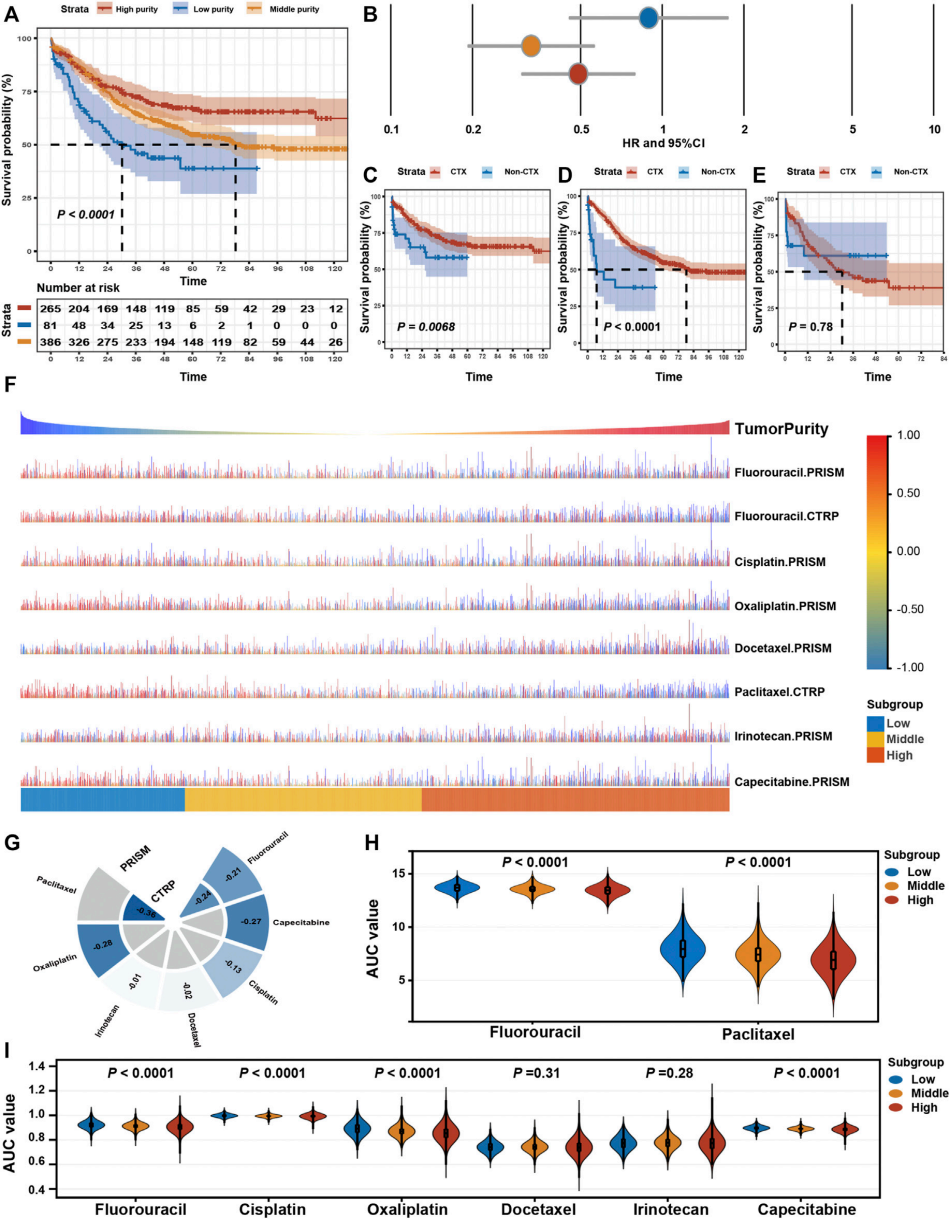
Drug response features of tumour purity for chemotherapy of gastric cancer. **(A)** Kaplan–Meier curves for overall survival (OS) among different purity subgroups of patients who received adjuvant chemotherapy. *p*-values were obtained using the log-rank test. The + symbols in the panels indicate censored data. **(B)** Subgroup analyses for adjuvant chemotherapy using the Cox proportional hazards regression model. The lines represent the 95% confidence intervals of the hazard ratios. **(C–E)** Kaplan–Meier curves for OS among patients who received adjuvant chemotherapy (CTX) and those who did not (non-CTX) in the **(C)** high-purity, **(D)** middle-purity and **(E)** low-purity subgroups. *p*-values were obtained using the log-rank test. The + symbols in the panels indicate censored data. **(F)** The predicted area under the curve (AUC) value of chemotherapeutic drugs for each patient. **(G)** The Spearman correlation coefficient between tumour purity and AUC values. **(H,I)** Differences in the predicted AUC value for chemotherapeutic drugs based on the **(H)** CTRP and **(I)** PRISM databases among the three subgroups. The upper and lower ends of the boxes represent the inter-quartile range of values. The horizontal width of the violin represents the data density. *p*-values were obtained via the Kruskal–Wallis test. CTX, adjuvant chemotherapy; non-adjuvant chemotherapy, non-CTX.

Furthermore, we determined whether varied purity levels were associated with differences in clinical benefit from adjuvant chemotherapy. Among patients who received adjuvant chemotherapy (*n* = 732), we found significant OS differences among three subgroups (Figures 6A,B). Adjuvant chemotherapy was associated with significantly increased OS and RFS rates in patients with GC in the high- and middle-purity subgroups (Figures 6C,D). However, no benefit from adjuvant chemotherapy was observed among patients with GC in the low-purity subset (Figure 6E).

Eventually, we assessed the value of GC purity in facilitating individualised chemotherapy regimens. We performed ridge regression to predict the drug susceptibility results for each sample (Figure 6F) using the CTRP and PRISM-derived drug response data. Spearman correlation analysis between the AUC value and GC purity was used to select agents with a significant correlation coefficient (Figure 6G). In addition, a differential analysis among the three subtypes was conducted to identify agents with lower estimated AUC values in each subgroup (Figures 6H,I). These analyses yielded two CTRP-derived compounds (fluorouracil and paclitaxel) and four PRISM-derived compounds (fluorouracil, capecitabine, cisplatin, and oxaliplatin). These compounds had lower estimated AUC values in the high-purity subgroup and a negative correlation with GC purity, suggesting that chemotherapy resistance to these five agents was observed in patients with GC with low tumour purity.

## 4 Discussion

GC tissues have a diverse mixture of tumour and nontumour cells within their microenvironment. Tumour purity has been recognised as a potential prognostic factor for GC (Aurello et al., 2017; Zhou et al., 2017; Ahn et al., 2018; Kemi et al., 2018). However, the purity level in previous studies was estimated by pathologists through visual evaluation, which could be affected by the sensitivity of histopathological characteristics, interobserver bias and variability in accuracy (Cohen et al., 2012; Smits et al., 2014). Moreover, because GC is a highly heterogeneous disease, analyses based on one or a few datasets inevitably lead to the neglect of tumour heterogeneity, a non-negligible factor for cancer biology and treatment (McGranahan et al., 2016). Therefore, previous single-centre studies with relatively small sample size limited the significance of their results. More importantly, previous studies failed to assess features other than clinical outcomes owing to the lack of different omic data.

Instead of the routine pathology-based estimation, we used the ESTIMATE algorithm, a computational method, in this study owing to its compatibility with RNA-seq and microarray transcriptome profiles, thus resulting in a more objective and accurate assessment (Aran et al., 2015). The extremely high tumour purity in various GC cell lines further suggested that the ESTIMATE algorithm has excellent robustness in calculating tumour purity in GC. In the present study, we integrated 2,259 GC cases from 13 different cohorts into a meta-data cohort and systematically investigated the role of GC purity. Owing to the benefits of the meta-data cohort with large sample size, tumour heterogeneity could be fully considered, which enhanced the reliability of our results and enabled us to reveal common key features of GC purity (Ricketts et al., 2018). We found that GC tissues from the same patient had a high concordance of purity level. Moreover, GC purity was strongly associated with clinical, biological and microenvironmental features. These findings suggest that tumour purity may be a patient-specific intrinsic characteristic of GC (Aran et al., 2015). Therefore, false interpretations owing to varied purity levels may negatively affect our understanding of GC biology and our ability to select the optimal treatment strategy.

For clinical features, consistent with the results of previous studies (Aurello et al., 2017; Zhou et al., 2017; Ahn et al., 2018; Kemi et al., 2018), low tumour purity was more likely to be found in the malignant phenotype and was associated with poor prognosis outcomes (OS and RFS). In addition, low tumour purity was strongly associated with mesenchymal, invasive and EMT phenotypes. Previous studies have suggested that malignant GC cells can recruit abundant surrounding cells within the TME and subjugate them to create a protective shield against immune attack (Silver et al., 2016). However, GC cells with limited invasive and metastatic features tend to form a solid bulk with less nontumour cell infiltration (Zhang et al., 2017). Therefore, the strong relationship between GC purity and clinicopathological and molecular factors may be partially attributed to their role in maintaining the balance between tumour and nontumour cells (Ying et al., 2011). Low tumour purity and correlated cellular heterogeneity may be responsible for the aggressive phenotype and poor prognosis of GC. Therefore, GC purity provides novel insights into estimating malignant phenotypes, thus explaining why most therapeutic strategies aimed purely against GC cells do not have an ideal outcome.

Furthermore, we determined that tumour purity is a robust independent prognostic indicator for GC. GC purity retained its prognostic relevance even after the classic clinicopathological prognostic factors were considered. These findings highlight the pivotal role of nontumour cells in the prediction of prognosis. Therefore, we integrated GC purity with other independent indicators to develop a prognostic nomogram. Compared with the TNM staging system, the nomogram showed superior validity and reliability in predicting survival time (OS and RFS), which further emphasised the requirement of considering GC purity in clinical management, especially for prognostic prediction.

Concerning biological features, distinct functional processes were the primary differential phenotype that resulted from varied GC purity levels. High tumour purity was associated with metabolism- and proliferation-related pathways. However, low tumour purity was associated with stromal- and immune-related pathways. For example, the IL6–JAK–STAT3 pathway, which is involved in the proliferation, survival, invasiveness and metastasis of tumour cells and suppresses the antitumour immune response in TME (Johnson, 2018), had high pathway activity in the low-purity group. The IL2–STAT5 signalling pathway, which is important for maintaining the development and function of regulatory T cells (Jones et al., 2020), was activated in the low-purity group. These results may explain the malignant phenotype and unfavourable prognosis of low-purity tumours.

Concerning the microenvironmental features, consistent with the finding that low GC purity was markedly associated with stromal and immune activation pathways, we found that tumour purity had a close relationship with the characteristics of cell infiltration in TME. Endothelial cells and fibroblasts were markedly enriched in low-purity tumours. Survival analyses revealed that the proportion of endothelial cells and fibroblasts presented negative prognostic value, partially explaining the unfavourable prognosis in the low-purity group. However, not all immune cells were substantially enriched in tissues with low GC purity. The number of antitumour cells, represented by CD8^+^ T cells and NK cells, may not increase in low-purity tumours, suggesting that these antitumour immune cells cannot infiltrate the established protective shield around GC cells (Zhang et al., 2020).

Eventually, we assessed the potential therapeutic effects of tumour purity in GC. The clinical data suggest that high GC purity can serve as an independent predictor for adjuvant chemotherapy benefits. Compared with those in the low-purity subgroup, patients in the high- and middle-purity subgroups could benefit more from adjuvant chemotherapy. Consistently, the drug response analysis revealed that the high tumour purity was positively correlated with chemotherapy sensitivity, suggesting that patients with high purity values are more sensitive to chemotherapy. Significant activation of proliferation- and metabolism-related pathways in high-purity tumours may induce considerable sensitivity to paclitaxel and fluorouracil. The low sensitivity of low-purity tumours to adjuvant chemotherapy is consistent with our observation that low-purity tumours contain abundant fibroblasts, which are associated with chemotherapy resistance (Yu et al., 2017). Low-purity tumours are also characterised by significant activation of the EMT signalling pathway. EMT has been reported to confer resistance to chemotherapy (Sale et al., 2019). Signalling pathways that regulate EMT, such as TGF-β and hedgehog pathways, are activated in low-purity tumours and correlated with chemotherapy resistance (Chen et al., 2021).

One of the main advantages of this study was the use of meta-data cohorts with large sample size and systematic analysis of tumour purity in multidimensional profiles. Our findings highlight the critical role of tumour purity in GC biology and clinical management. However, the present study had several limitations, such as the retrospective nature of clinical data. This study focussed on analysing the association of clinical and molecular factors with the whole microenvironment (tumour purity) without referring to specific cell types. Therefore, further in-depth studies should be conducted to interpret the effects of specific cell types on TME and assess their relationship with GC cells.

In conclusion, this study highlights that GC purity is closely associated with clinical, biological, TME and drug-response features. Therefore, ideal clinical management of GC should focus on not only the properties of tumour cells but also non-tumour components. A comprehensive evaluation of tumour purity in individual patients with GC can help to elucidate the complex role of the GC microenvironment and provide novel insights into individualised treatment regimens.

## Data Availability

The datasets presented in this study can be found in online repositories. The names of the repository/repositories and accession number(s) can be found in the article/Supplementary Material.

## Supplementary Material

Supplementary Figure 1 Tumour purity in gastric cancer (GC) cells and tissues. **(A)** Principal component analysis (PCA) score plot for the expression profile of the 13 datasets before (left) and after (right) batch effect removal. **(B)** Spearman correlation analysis of purity value before and after chemotherapy. **(C)** Spearman correlation between purity value and age.

Supplementary Figure 2 Prognostic nomogram based on tumour purity and clinical factors for gastric cancer. **(A)** Prognostic nomogram for overall survival (OS). **(B)** Prognostic nomogram for recurrence-free survival (RFS).

Supplementary Figure 3 Weighted correlation network analysis (WGCNA) for tumour purity. **(A)** Evaluation of soft-thresholding power. **(B)** Hierarchical dendrogram of the co-expression modules identified using WGCNA. **(C)** The biweight midcorrelation correlation between module membership and gene significance for the yellow, blue, red and turquoise modules.
