# Supplementary figures and images for: Comprehensive Characterization of Tumor Purity and Its Clinical Implications in Gastric Cancer
Source: Front Cell Dev Biol. 2022 Jan 10;9:782529. doi: 10.3389/fcell.2021.782529 (PMC8784737; doi:10.3389/fcell.2021.782529)

A

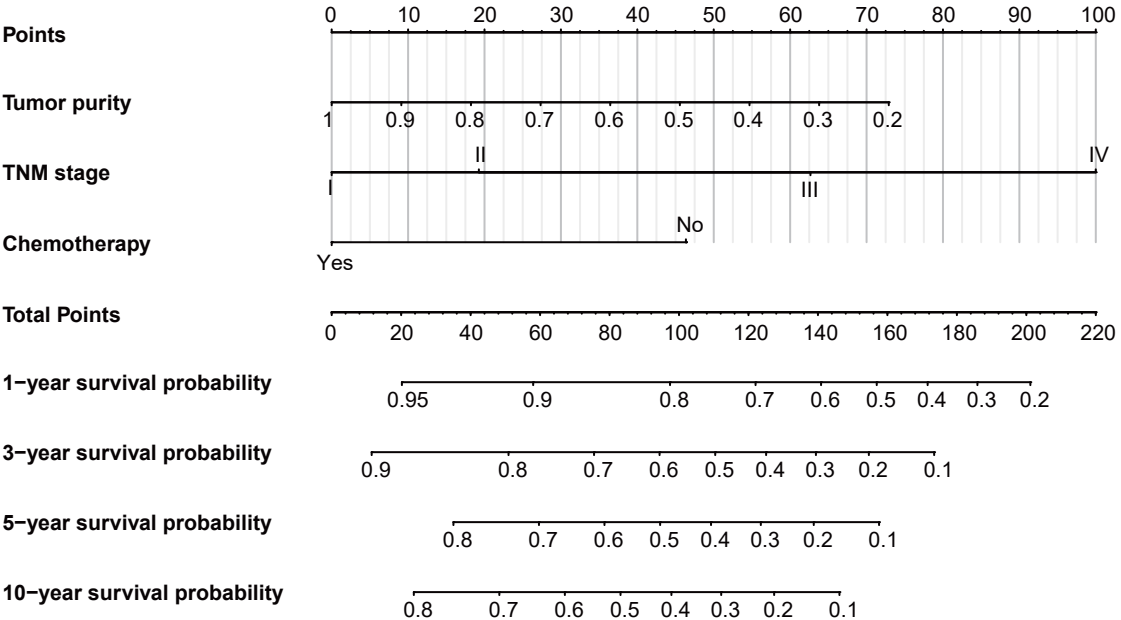

B

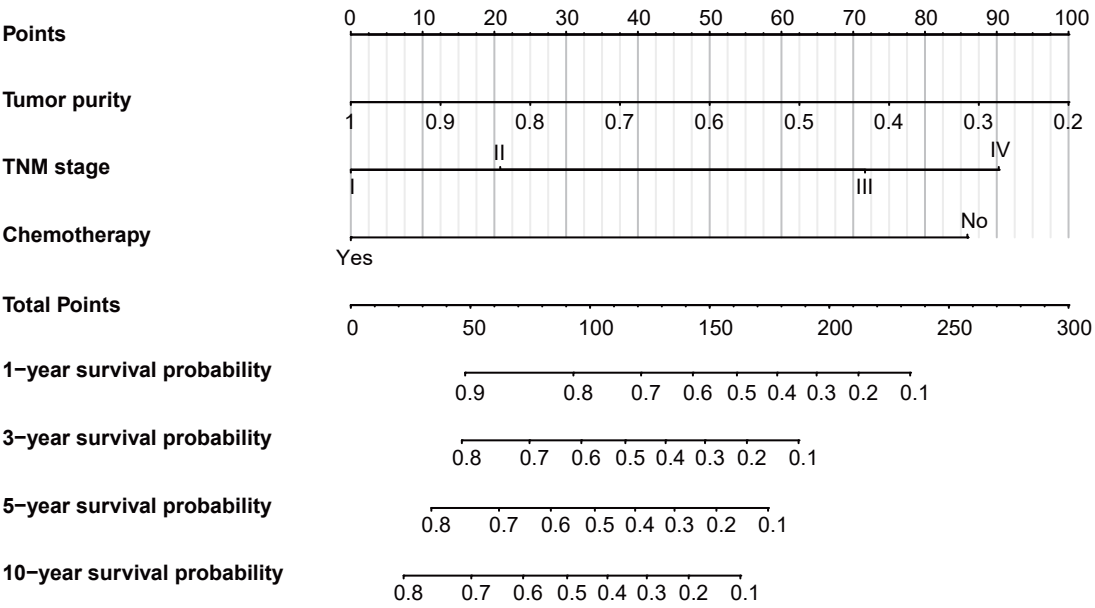

Supplement: Supplementary file 1 [file DataSheet2.PDF]

**A**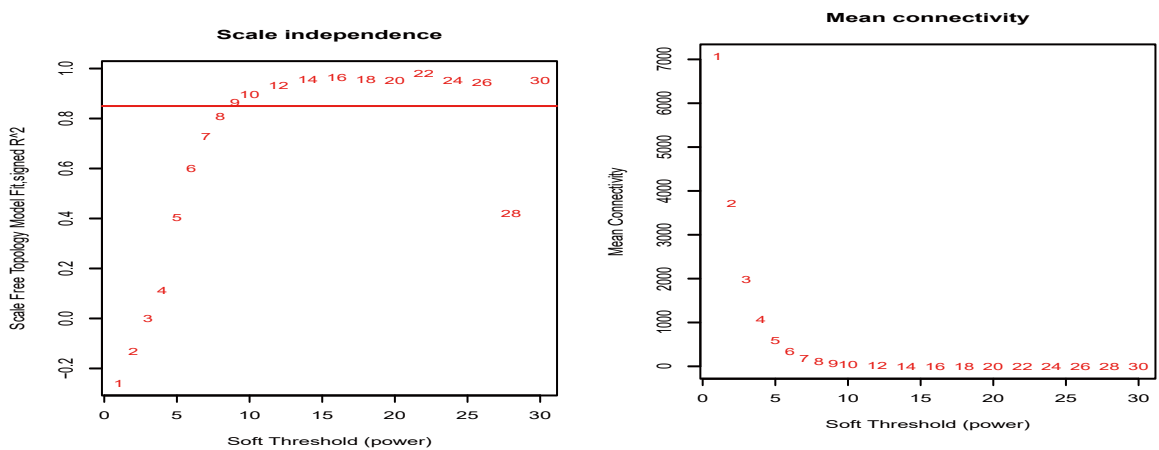**B**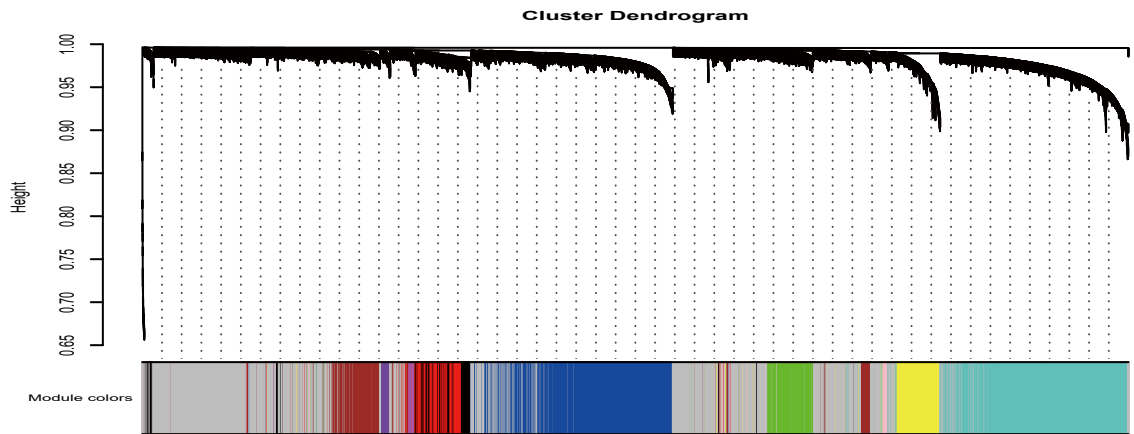**C**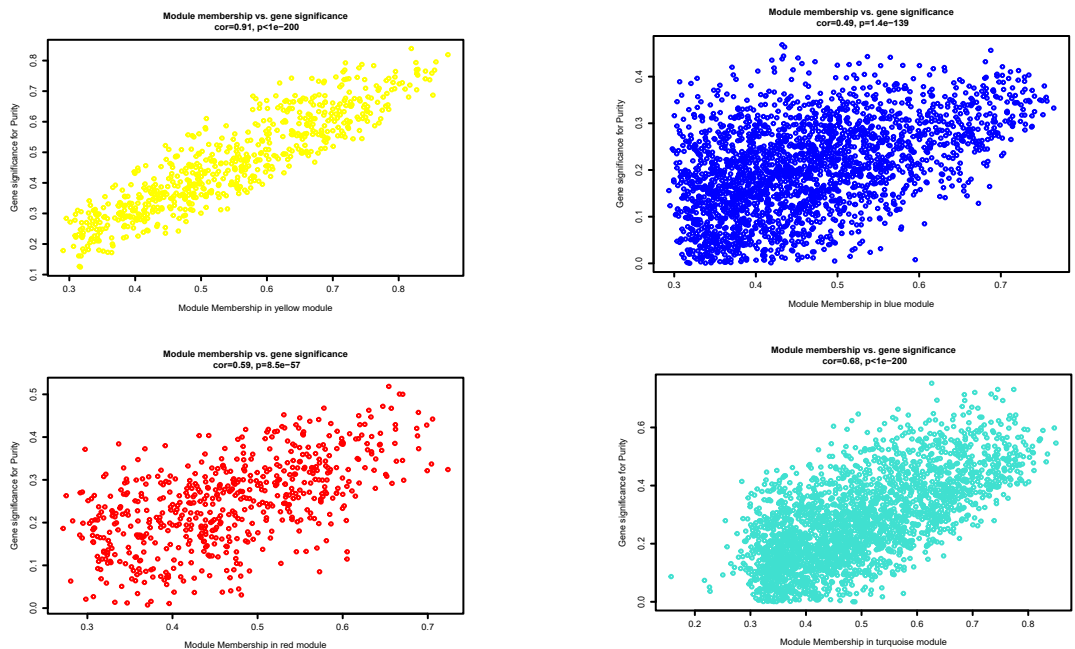

Supplement: Supplementary file 2 [file DataSheet3.PDF]

**A**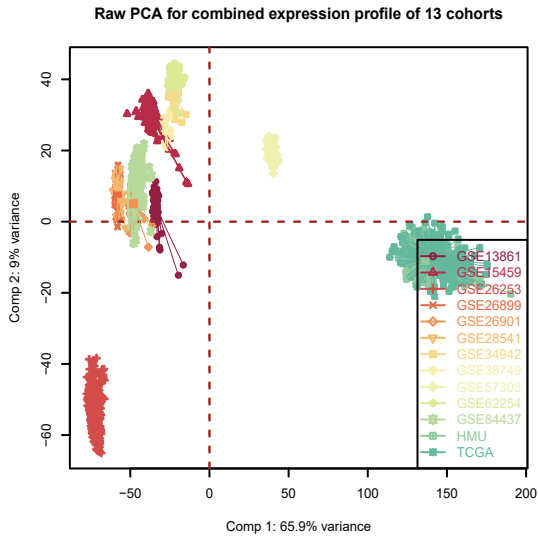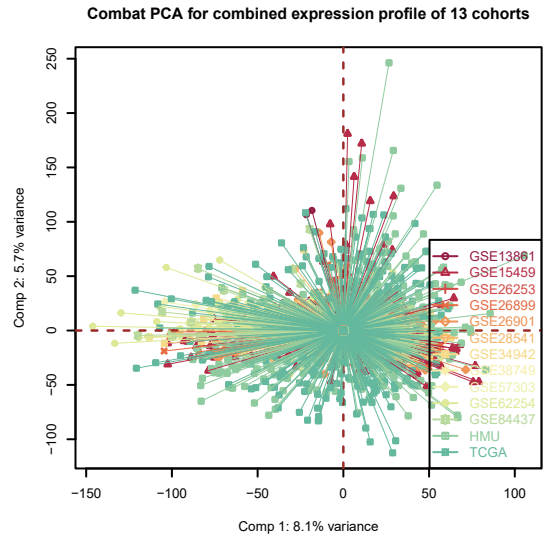**B**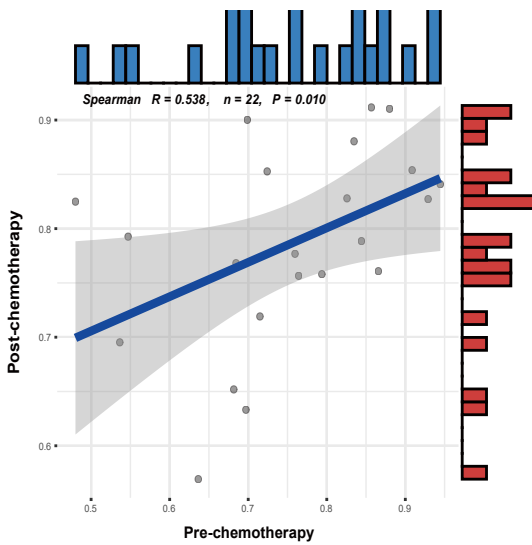**C**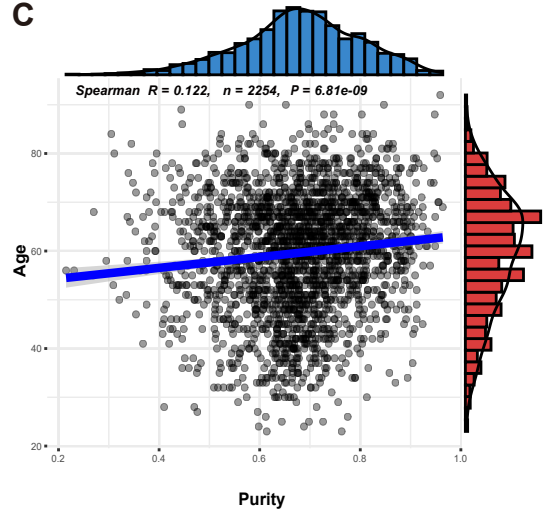

Supplement: Supplementary file 3 [file DataSheet1.PDF]
